# Clinical Application and Efficacy of Silver Drug in Ophthalmology: A Literature Review and New Formulation of EYE Drops with Drug Silver (I) Complex of Metronidazole with Improved Dosage Form

**DOI:** 10.3390/biomedicines9020210

**Published:** 2021-02-19

**Authors:** Arleta Waszczykowska, Dominik Żyro, Justyn Ochocki, Piotr Jurowski

**Affiliations:** 1 Department of Ophthalmology and Vision Rehabilitation, Medical University of Lodz, Zeromskiego 113, 90-549 Łódź, Poland; piotr.jurowski@umed.lodz.pl; 2 Department of Bioinorganic Chemistry, Medical University of Lodz, Muszyńskiego 1, 90-151 Łódź, Poland; dominik.zyro@umed.lodz.pl (D.Ż.); justyn.ochocki@umed.lodz.pl (J.O.)

**Keywords:** silver (I) complex, ophthalmic diseases, ocular rosacea, ointment, eye drops, drops stabilization

## Abstract

The use of silver preparations in medicine is becoming increasingly popular. The basic aim of this evaluation was to review the literature on the clinical (in vivo) and antibacterial potential of silver preparations in ophthalmic diseases. The second goal was to summarize the results of experimental research on the use of silver preparations in ophthalmology. The third objective was to present a method for stabilizing eye drops containing silver (I) complex. Analysis of the pH stability of the silver (I) complex with metronidazole in the prepared dosage form (eye drops) was carried out. Most silver preparations are clinically used for topical application. Few experimental results indicate the usefulness of intraocular or systemic administration of silver (I) preparations as an alternative or additional therapy in infectious and angiogenic eye diseases. The development of a new formulation increases the stability of the dosage form. New forms of silver (I) products will certainly find application in the treatment of many ophthalmic diseases. One of the most important features of the silver (I) complex is its capacity to break down bacterial resistance. The new eye drops formula can significantly improve comfort of use. Due to their chemical nature, silver (I) compounds are difficult to stabilize, especially in the finished dosage form.

## 1. Introduction

Tackling the growing problem of drug resistance to antibiotics and chemotherapeutics is a challenge of modern medicine. Every year, despite advanced methods of treatment, growing levels of health protection and increasing public awareness of drug abuse, the number of deaths caused by strains of bacteria resistant to antibiotics increases [1]. Therefore, new compounds with a potential antimicrobial effect are being sought, and interest in the use of precious metals in medicine as an alternative in the fight against infections is returning. Recently, research on elements such as copper, zinc, titanium, nickel, magnesium, gold and silver has brought hope for effective alternative methods in the fight against infections [1,2,3]. Among these metals, silver has been especially widely used in medicine and has a well-documented antimicrobial effect against Gram-positive and Gram-negative bacteria, fungi, protozoa and viruses [4].

In medicine, silver is used as a metal and as salts and nanoparticles (colloidal) from a chemical viewpoint [5]. Silver owes its antibacterial and antifungal properties to its ionic form of Ag(I) only, which, however, is unstable and can be easily inactivated by inappropriate complexation and precipitation, or transform into a metallic form of Ag(0) without healing properties [6].

Silver metal continuously releases small amounts of ions, which act as an antibacterial at the metal surface [5]. Oxidation to the Ag(I) ion is a slow process under normal conditions and leads to low effective silver concentrations. Therefore, metallic silver has been used in alloys with which implants or sutures are coated [7,8].

Silver (I) salts, containing the ionic, microbiologically active form, are divided into practically insoluble (silver (I) sulfide) and well-soluble (silver (I) nitrate). The high solubility of silver (I) salts leads to a high local silver concentration and, thus, high antibacterial activity but high toxicity as well. The solubility and toxicity of silver (I) salts are dependent on many environmental factors; e.g., they vary according to pH. Hence, any medicinal product containing silver (I) salts requires careful clinical studies assessing the actual concentration of silver (I) ions [9].

Nanoparticulate (colloidal) silver synthesis involves the reduction of a soluble silver (I) salt by a reductant such as citrate, glucose, ethylene glycol or sodium borohydride in the solution phase [10]. The addition of stabilizing compounds, which prevent growth and aggregation of the formed silver nanoparticles, plays a decisive role [9]. Repeated synthesis of silver nanoparticles in laboratory conditions is difficult and depends on, among other things, concentrations, the reduction agent, temperature and the presence of additives. Additionally, the morphology of the created particles is sometimes not stable over time. Often, synthesized silver nanoparticles tend to aggregate after a few hours or days if the colloidal stability is insufficient [5,10].

Alternatively, silver nanoparticles (Ag-NPs) can also be synthesized biologically using microbes such as *Bacillus subtilis* and *B. licheniformis* (Gram-positive bacteria), *Escherichia coli* (Gram-negative bacteria), fungi, yeasts and viruses [6,11]. In addition, due to the richness of alkaloids, saponins, tannins, vitamins, phenols and terpenoids in organic components, Ag-NPs synthesis uses plants, plant products and algae as biological samples and reducers, providing an inexpensive, one-step synthesis procedure [12]. Novel noble silver nanoparticles are attractive as antimicrobial agents for their surface functionalization versatility and their capacity to cleave disulfide bonds. Ag-NPs interact with bacteria, fungi and viruses in a shape-dependent manner. As the particle size decreases, the percentage of surface atoms increases, creating many unsaturated bonds due to the lack of adjacent atoms. As a consequence, Ag-NPs have unstable atoms with high surface energy. This type of structure provides many contact adsorption sites and reaction points that can be further modified [13].

Metallic silver is typically inert, but in the presence of tissues, when implanted, it becomes ionized due to the presence of oxygen, moisture and body fluids, releasing biologically active silver ions (Ag^+^) that bind to thiol groups (-SH), anionic ligands of proteins and cell membranes of bacterial cells [14]. The basis of the antimicrobial effect of silver is the ability of Ag (I) to penetrate cell walls of bacteria through pinocytosis, causing an increase in cellular oxidative stress in microbes—denaturing and inactivating proteins as well as metabolic enzymes—that leads to growth arrest [14,15]. Ionic silver (I) also has the ability to bind to the microbial genome (DNA or RNA) through denaturation, which inhibits replication and the possibility of microbial multiplication [16].

The most recent discovery of Ag-NPs as biocides involves their potency as antiviral agents against viral infectious diseases, such as SARS-CoV, influenza A/H5N1, influenza A/H1N1, herpes simplex virus types 1 and 2, human parainfluenza virus type 3, dengue virus, HIV-1, hepatitis B virus and new encephalitis viruses. The exact mechanism of Ag-NPs as antiviral agents has not yet been fully explained [17]. Generally, silver nanoparticles are capable of reducing viral infectivity, probably by blocking interaction of the virus with the cell, which might depend on the size and zeta potential of the silver nanoparticles [18]. In vitro studies have shown the effectiveness of silver NPs modified with oseltamivir in reducing influenza glycoproteins and preventing DNA fragmentation, condensation of chromatin and caspase-3 functions to effectively reduce H1N1 infection [18,19,20]. The latest research revealed suppression of human parainfluenza 3 (HPIV-3) replication, possibly due to the blocking function of the cell-virus by leveraging Ag-NPs [21].

The cytotoxic potential of silver nanoparticles or silver (I) ions from silver (I) azole complexes has been reported in different cancer cell lines, including A549 (lung cancer) [22], MCF-7 (breast cancer) [23], HT29 (colon cancer) [24], HeLa (cervical cancer) [25], HepG2 (liver cancer) [26], PANC-1 and 1.2B4 (human pancreatic cancer) [27].

The anti-inflammatory effects of silver (I) nitrate or nanocrystalline silver have been recognized experimentally in wound care and the treatment of allergic contact dermatitis and ulcerative colitis [28,29,30]. Experimental studies have shown a decrease in inflammation following the application of nanocrystalline silver, which was associated with lymphocyte apoptosis, reduced expression of proinflammatory cytokines and decreased activity of gelatinase [31].

In our work, we review data on the use of silver preparations in ophthalmology concerning indications for treatment, its results and complications. Additionally, we summarize the results of experimental research on the application of silver (I) compounds in ophthalmology and present a method of stabilizing eye drops containing a silver (I) complex.

## 2. Silver in Ophthalmology

### 2.1. History of Silver as an Antibacterial Agent

The first mention of the use of silver in medicine comes from ancient times. Hippocrates probably already used silver preparations for the treatment of ulcers and to promote wound healing. Soluble silver (I) compounds, such as silver (I) nitrate, were first used empirically as a blood-purifying agent and date back to 702–705 AD [31]. Later, silver (I) salts were used as an antimicrobial agent to treat infectious diseases, including syphilis and gonorrhea, brain infections, epilepsy, mental illness, nicotine addiction and gastroenteritis [32].

The widest use of silver in medicine took place in the 1880s. In these years, the first silver plate was implanted during cranioplasty, and the use of eye drops with silver (I) nitrate solution was started to prevent childhood blindness and reduce gonococcal ophthalmia neonatorum [33]. Mandatory ophthalmic prophylaxis in newborns with drops of silver (I) nitrate, as in Credé’s method, was accepted in many countries throughout the world until the 1970s, and in some areas it still remains a routine part of perinatal care today [34,35]. Over the years, the use of silver (I) preparations in ophthalmology has been significantly extended to treat corneal ulcers, interstitial keratitis, blepharitis and dacryocystitis [36].

### 2.2. Toxicology

The toxicology of silver is not well documented, and much of the available information concerning the release of Ag (I) from medical devices and other products intended for human use has been ambiguous. The few publications with experimental results in animal models have also been inconsistent. However, although they are insufficient in predicting human risk from silver exposure, they do provide relevant information on cytotoxicity, intracellular management of Ag (I) and excretion routes [37].

Studies of silver metabolism indicate that soluble silver (I) compounds are more easily absorbed as a result of their ability to bind to proteins, DNA and RNA. Soluble silver (I) compounds can be quickly absorbed into the bloodstream, deposited throughout the body and then reduced by light to metallic silver [38]. Acute symptoms of overexposure to silver (I) nitrate are decreased blood pressure, diarrhea, stomach irritation and decreased respiration. Chronic symptoms from prolonged intake of low doses of silver (I) salts are fatty degeneration of the liver and kidneys and changes in blood cells [39]. Soluble silver (I) compounds are also capable of accumulating in small amounts in the brain and in muscles [40].

The literature reports that long-term inhalation or ingestion of soluble silver (I) compounds or colloidal silver may cause necrosis of conjunctival epithelial cells, argyria and/or argyrosis [40,41,42]. Moreover, irritation, conjunctival scars, corneal opacity and symblepharon have been noted [43]. In clinical practice, the diagnosis of eye argyrosis may not be easy due to the rarity of this disease. Differential diagnosis should include other keratopathies (e.g., pre-Descemet dystrophy and X-linked ichthyosis) as well as other causes of abnormal eye pigmentation, such as malignant melanomas, deposition of heavy metals (iron and copper) or drugs (ciprofloxacin and amiodarone) [44,45].

The recent results of experimental studies did not show significant toxic effects of Ag-NPs and AgNO_3_ in doses up to 1 mg kg-1 of rat body weight. The distribution of Ag in organs was similar in both studied groups of treated rats. The total Ag content in organs was significantly lower in rats treated with Ag-NPs, but in this group, it was found to be more toxic in terms of biochemical and hematological parameters than in rats treated with AgNO_3_ [46]. Ag-NPs probably caused reactive oxygen species and oxidative damage, which would confirm oxidative stress as an additional possible mechanism of Ag-NPs’ toxicity [46]. Other studies have shown lower toxicity of Ag-NPs compared to AgNO_3_, which was attributed to the coatings that stabilize nanoparticles and thus reduce their toxicity [47].

## 3. Metronidazole in Ophthalmology

Metronidazole (2-methyl-5-nitroimidazole-1-ethanol), a 5-nitroimidazole derivative, has been established as the drug of choice for the treatment of several systemic protozoal diseases, such as amoebiasis and anaerobic bacterial infections [48].

The mechanism of action of metronidazole is based on the production of compounds inside cells that destroy microbial DNA. The drug also has an anti-inflammatory and immunomodulatory effect by directly [49] influencing T lymphocytes and causes changes in the function of neutrophil cells, inhibiting the production of reactive oxygen species [50]. In addition, metronidazole may have acaricidal activity, as it is degraded in vivo into at least five metabolites with potent biological activity (e.g., its 2-hydroxymethyl derivative is 1/3 to 10-times more active as an antibacterial agent than metronidazole itself) [51].

There are only a few reports on systemic metronidazole therapy in ophthalmology. Metronidazole is used after penetrating orbitocranial trauma [52], in autoimmune uveitis [53] and *Demodex* eradication [54,55]. The results of studies on the efficacy of oral metronidazole alone for the treatment of *Demodex folliculitis* skin lesions are inconclusive. Some have shown a marked, rapid and lasting reduction of the inflammatory picture and *Demodex* population [54]; others have been implicated in the improvement of inflammation but without affecting the *Demodex folliculitis* population [55].

Since *Demodex* mites are present in healthy eyelids, toxic or very effective systemic treatment may not be necessary. No clinical side effects or liver toxicity after metronidazole treatment have been observed in studies. However, hypersensitivity reactions are more frequent in systemic compared to topical treatments. Serious reactions have been observed using metronidazole in other parasitic infections, such as the Mazzotti reaction (tachycardia, hypotension, arthralgias, edema and abdominal pain), Steven–Johnson and Lyell diseases, fatal encephalopathy, increased INR (International Normalized Ratio) with hemorrhage, decreased leukocyte count, anemia, hepatitis, elevated liver enzymes and increased bilirubin levels [56,57], as well as itching, irritation and dryness of the skin [58].

Topical preparations of metronidazole have been used since 1980 in the form of ointments to treat eyelid infections in the course of acne rosacea [58]. Recently, metronidazole has also been used successfully as an antiprotozoal drug in the treatment of *Acanthamoeba* keratitis, and so far the use of ophthalmic drops with metronidazole in humans for the treatment of amoebic corneal inflammation [48] and ocular rosacea has been described [59]. Experimental studies have proven that topical administration of metronidazole reduces corneal neovascularization [60]. To the best of our knowledge, there are no reports in the literature on the treatment of ophthalmic complications of rosacea with metronidazole drops.

## 4. Miconazole in Ophthalmology—Fungal and Fungal/Bacterial Eye Infections

Miconazole nitrate is an antifungal and antibacterial drug belonging to the imidazole derivatives. It inhibits the biosynthesis of ergosterol in fungal cells, which causes a change in the composition of cell membrane lipids, causing the death of fungal cells. It has a fungicidal, bactericidal and bacteriostatic effect. It has an effect on yeasts, dermatophytes, radiophytes and Gram-positive bacteria [61]. Moreover, it has been proven that topically applied miconazole might be a therapeutic option for Gram-positive aerobic bacteria in skin infections, even against those strains that are resistant to antibiotics [62].

Antifungal drugs are usually administered intravenously, and in miconazole’s systemic use, anemia, nausea, fever and chills have been reported [63]. The main problem with systemic administration is to obtain sufficiently high intraocular concentrations, especially in diseases where high concentrations are required [64]. The ocular penetration of antifungal drugs, like most antibiotics, is poor. In the case of amphotericin B, many so-called saprophytic fungi demonstrate high resistance to it in vitro, many times higher than the highest achievable blood concentration [65]. Therefore, other modes of antifungal medication administration have been reported in ophthalmology: topical (as drops or ointment), subconjunctival and intravitreal injections [64].

High topical miconazole efficacy, comparable to fluconazole, has been proven in treating *Candida albicans* keratomycosis [66] as well as *Alternaria*, *Rhodotorula* and *Aspergillus* corneal ulcers [67].

Combined systemic and topical therapy has been shown to be very effective in the treatment of *Acanthamoeba* keratitis [68] and *Streptomyces gougerotii* dacryocystitis [67].

Studies on the toxicity of topical antifungal drug solutions have indicated the superiority of miconazole and flucytosine in the treatment of corneal inflammation, which did not delay healing of pathological changes in the regenerating corneal epithelium [69].

Experimental studies have shown the safety of intravitreally administered miconazole not exceeding 40 μg for fungal endophthalmitis [70].

## 5. Application of Silver Compounds in Ophthalmology

Nowadays, silver metal and silver nanoparticles are used as gels or films for medical device coatings and the reduction of bacterial adhesion to the surfaces of implants [59]. In ophthalmology, the use of nano-silver (nAg) for covering corneal prosthetic devices (KPros) has been sought to prevent *Pseudomonas aeruginosa* and *Staphylococcus aureus* biofilm formation as a protection against perioperative and early postoperative infections [71]. Intrastromal administration of highly biocompatible gelatin-capped silver nanoparticles (G-Ag NPs) has been a promising, dual-functional (antimicrobial and antiangiogenic) nanotherapy for the treatment of *Staphylococcus aureus*-induced bacterial keratitis in rabbits [72].

New applications of silver in ocular disease diagnosis and treatment are still being sought. The high usefulness of silver-amplified immunochromatography for detection of adenoviral conjunctivitis has been demonstrated [73].

It has also been reported that Ag-NPs are a potential alternative, and may even be better than mitomycin C, as an adjunctive therapy in glaucoma surgery to reduce fibroblast proliferation and bleb fibrosis after trabeculectomy [74].

The strong therapeutic effect of biologically synthesized silver nanoparticles against vascular endothelial growth factor (VEGF) has been proven. Ag-NPs, as inhibitors of tyrosine kinase Src and AKT/PI3K pathways, are able to inhibit further routes of angiogenesis from proliferative diabetic retinopathy and age-related macular degeneration in rodent eye models and, therefore, appear to be a promising treatment for many retinal diseases in humans [6,75,76].

The incorporation of Ag-NPs into silicone-hydrogel contact lenses allows for significant inhibition of bacterial growth and reduction of biofilm formation, with additional reinforcement of some of the mechanical properties [77].

## 6. Silver (I) Complexes with Azole Derivatives in Ophthalmology

### 6.1. Silver (I) Complex with Metronidazole in Ophthalmology

Research on the use of new silver preparations combined with other active substances in ophthalmology is developing much more vigorously [78]. In our research group, we have conducted numerous studies that showed greater stability of metal ion complexes compared to metal ion salts [27] and lower toxicity due to the possibility of using lower concentrations of silver ions in complexes [78].

The widest and best-known use of silver in medicine has been in combination with sulfadiazine (AgSD), where it becomes a topical antibacterial agent for the treatment of burns [79] and fungal keratitis [80,81]. The action of AgSD also demonstrates strong antibacterial potential against *E. coli*, *Staph. aureus*, *Klebsiella* sp. and *Pseudomonas* sp. [16].

In terms of biological performance, studies in vitro, as well as in vivo, on retinal pigment epithelium with endophthalmitis in mouse and rabbit models confirm the cellular biocompatibility and antibacterial function of silver complex nanomaterials. The addition of photodynamic therapy with Ag-NPs as well as AuAgCu_2_O-bromfenac sodium nanoparticles allows the antibacterial effect to be strengthened against *Escherichia coli*, *Staphylococcus aureus* and methicillin-resistant *S. aureus* for synergistic treatment of post-cataract surgery endophthalmitis [82,83].

Ag-NPs conjugated with oleic acid or vildagliptin exhibit antiacanthamoebic activity that can be therapeutically applied against *Acanthamoeba castellanii*, an opportunistic pathogen that is associated with blinding eye keratitis and a rare but fatal central nervous system infection [84,85].

In our previous paper, we described, for the first time in the literature, the action of a metronidazole complex with well-soluble silver (I) salts in the form of drops and ointment in the treatment of ocular rosacea [86]. The use of a well-soluble silver (I) complex with metronidazole reduced the side effects of silver (I) nitrate used alone and the costs and complications of standard antibiotic therapy. The use of two clinically proven drugs and combining them into a complex compound does not only result in additive synergy. As we have shown in our previous work, the action of the complex compound overcomes bacterial resistance [87].

**Figure 1:**
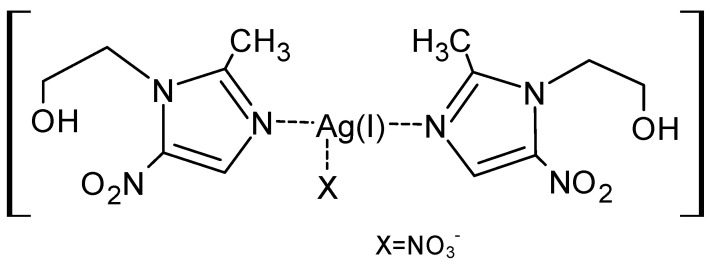
Silver (I) nitrate complex with metronidazole [Ag(MTZ)_2_]NO_3_.

**Scheme 1:**
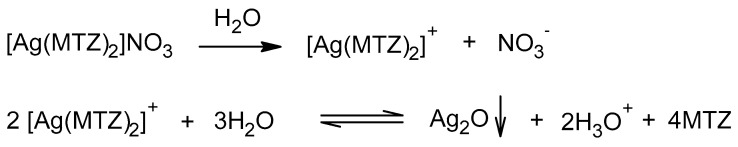
Dissociation of silver (I) nitrate complex with metronidazole and cation [Ag(MTZ)_2_]^+^ hydrolysis.

Silver (I) nitrate is a salt of a strong acid and is subject to hydrolysis in aqueous solutions. The products of hydrolysis are protons that lower the pH of the solution. Similarly, a complex of metronidazole with silver (I) (Figure 1) in the form of a nitrate salt undergoes hydrolysis over time (Scheme 1).

An important feature of eye drops is their compatibility with the pH of tear fluid, which is around 7.0–7.4. The currently recommended pH of eye drops is in the range of 3.5–8.5 [88,89]. However, a pH that is too low causes discomfort for the patient through a burning sensation or stinging.

**Scheme 2:**
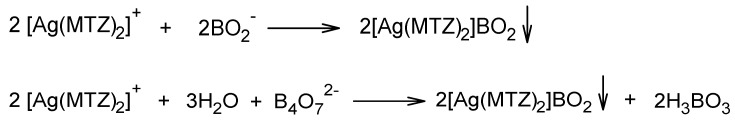
Precipitation of sparingly soluble borate salt of the silver (I)–metronidazole complex compound.

**Scheme 3:**
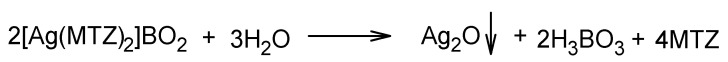
Decomposition of borate salt of the silver (I)–metronidazole complex.

**Scheme 4:**
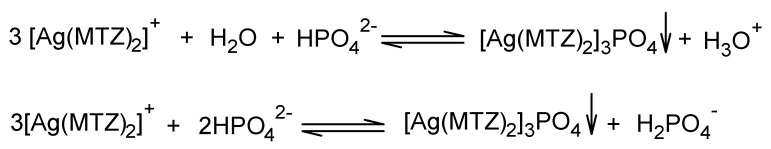
Precipitation of sparingly soluble phosphorate salt of the silver (I)–metronidazole complex compound.

The desired pH can be achieved by adding a buffer solution. Buffer solutions added to eye drops provide the required pH and isotonic properties. The reaction uses isotonic phosphate buffers to obtain a pH in the range of 6.0–8.0 and borate buffers to obtain a pH of 5.0–10.6. This is not possible with silver salts and silver complexes. Furthermore, reactions involving precipitation of very sparingly soluble silver (I) salts, such as borates (Scheme 2 and Scheme 3) or phosphates (Scheme 4), which do not have therapeutic properties, result in the solution becoming a suspension with particles that can irritate the eye.

The appropriate pH can be achieved without risking the precipitation of silver (I) hydroxide by adding sodium hydroxide, which then decomposes into silver (I) oxide. Sufficient sodium hydroxide binds excess protons in the drop solution and allows the pH to be raised to the desired level.

**Figure 2:**
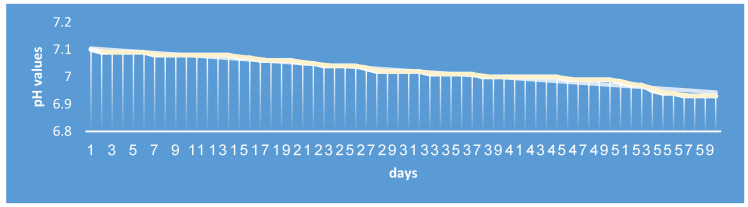
Change in the pH value of the drops during the storage period.

**Figure 3:**
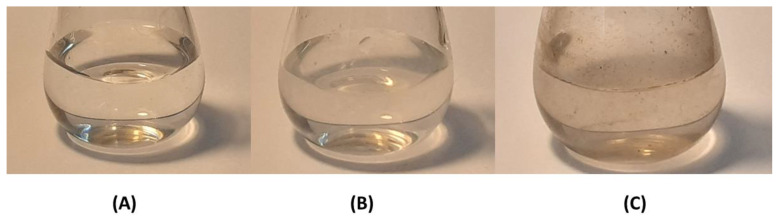
Eye drops solution on the first day (**A**) and 6 months after preparation, stored in a refrigerator (**B**) and at room temperature (**C**).

Excess H_3_O^+^ ions are bound by OH^−^ anions, which are formed as a result of complete dissociation of sodium hydroxide. The equilibrium state is established at pH = 6.9–7.1. The solution remains colorless and clear, and [Ag(MTZ)_2_]^+^ ions are present. The period of use of the manufactured product for individual patients was 1 month from the moment of opening the bottle, which is the generally accepted rule for eye drops and pharmaceutical standards all over the world. The form of the drug was produced according to an ex tempore pharmacy recipe. The dosage form prepared in the formulation was stored and evaluated for change in pH, clarity and transparency over 2 months. The pH did not change significantly during this time in storage. The pH value changed during storage to a minimum value of 6.9 (Figure 2). After 6 months of storage at room temperature, silver (I) oxide began to appear. Storing the drops at 2–8 °C in a refrigerator did not produce such results (Figure 3).

### 6.2. Miconazole Silver (I) Compounds as a Potential Candidate in the Fight against Mixed Infections in Ophthalmology

Study results showed that miconazole silver (I) compounds have promising therapeutic potential, can be used as antifungal, antibacterial, and anticancer agents and have stronger antibacterial and anticancer properties than free ligands [90,91]. These complexes may be considered as a therapeutic option in infections caused by fungal strains as well as by bacterial strains that are resistant to antibiotics. Recently, we reported an easy and convenient method for the synthesis of silver (I) complexes using miconazole and silver (I) nitrate and silver (I) perchlorate [26].

## 7. Conclusions

The antimicrobial effectiveness of silver preparations in ophthalmic diseases has been documented by many researchers. Complex silver (I) compounds seem to be a promising alternative to standard therapy and are, therefore, also considered as new generation antibiotics. Most silver (I) preparations are clinically used for topical applications. Few experimental results indicate the usefulness of intraocular or systemic administration of silver (I) preparations as an alternative or additional therapy in infectious and angiogenic eye diseases. New forms of silver (I) products will certainly find application in the treatment of many ophthalmic diseases. One of the most important features of the silver (I) complex is its capacity to break down bacterial resistance. It is very helpful to maintain the appropriate characteristics of the dosage form, e.g., pH and chemical, physical and pharmaceutical stabilities.

## Abbreviations

Ag-NPs	Silver Nanoparticles
AgSD	Silver Sulfadiazine
DNA	Deoxyribonucleic Acid
G-Ag NPs	Gelatin-capped Silver Nanoparticles
HIV-1	Human Immunodeficiency Virus 1
HPIV-3	Human Parainfluenza Virus 3
INR	International Normalized Ratio
MIC	miconazole
MTZ	metronidazole
nAg	nano-silver
RNA	Ribonucleic Acid
SARS-CoV	Severe Acute Respiratory Syndrome Coronavirus
VEGF	Vascular Endothelial Growth Factor
